# Humoral and cellular immune response after severe acute respiratory syndrome coronavirus 2 messenger ribonucleic acid vaccination in heart transplant recipients: An observational study in France

**DOI:** 10.3389/fmed.2022.1027708

**Published:** 2022-10-26

**Authors:** Alice Casenaz, Sandrine Grosjean, Ludwig-Serge Aho-Glélé, Jean-Baptiste Bour, Christelle Auvray, Catherine Manoha

**Affiliations:** ^1^Virology Laboratory, Department of Microbiology, Dijon Bourgogne University Hospital, Dijon, France; ^2^Department of Anaesthesiology and Critical Care Medicine, Dijon Bourgogne University Hospital, Dijon, France; ^3^Epidemiology and Infection Control Unit, Dijon Bourgogne University Hospital, Dijon, France

**Keywords:** SARS-CoV-2, heart transplant recipient, mRNA vaccine, BNT162b2, T cell, smoking, natural infection, antibody

## Abstract

**Introduction:**

Heart transplant (HT) recipients have a high risk of developing severe COVID-19. Immunoglobulin G antibodies are considered to provide protective immunity and T-cell activity is thought to confer protection from severe disease. However, data on T-cell response to mRNA vaccination in a context of HT remains limited.

**Methods:**

In 96 HT patients, a IFN-γ release assay and an anti-Spike antibody test were used to evaluate the ability of SARS-CoV-2 mRNA vaccines to generate cellular and humoral immune response. Blood samples were collected few weeks to 7 months after vaccination. Multiple fractional polynomial and LASSO regression models were used to define predictors of T-cell response.

**Results:**

Three to five months after vaccination, three doses of vaccine induced a positive SARS-CoV-2 T-cell response in 47% of recipients and a positive humoral response in 83% of recipients, 11.1% of patients remained negative for both T and B cell responses. Three doses were necessary to reach high IgG response levels (>590 BAU/mL), which were obtained in a third of patients. Immunity was greatly amplified in the group who had three vaccine doses plus COVID-19 infection.

**Conclusion:**

Our study revealed that T and B immunity decreases over time, leading us to suggest the interest of a booster vaccination at 5 months after the third dose. Moreover, a close follow-up of immune response following vaccination is needed to ensure ongoing immune protection. We also found that significant predictors of higher cellular response were infection and active smoking, regardless of immunosuppressive treatment with mycophenolate mofetil (MMF).

## Introduction

Solid-organ transplant recipients are at high risk of severe COVID-19, essentially because they are chronically immunosuppressed and frequently suffer from comorbid disease (1, 2). HT patients with SARS-CoV-2 infection frequently require hospitalization (42.5%) (3), and their mortality rate is higher than that of immunocompetent individuals (1, 3, 4).

In addition to high rates of complications and mortality from COVID-19 infections, the pandemic has complicated the transplant process. It added new questions regarding donor and recipient screening, potential exposure of recipients to a greater risk of immunosuppression and decision to transplant the patient. Moreover, the clinical implications of COVID-19 infection may differ depending on the type of organ transplanted and the recipient’s comorbidities which further impact decisions to pursue transplantation (5, 6). Solid-organ transplant recipients are among the groups for whom vaccination is a priority. The seroprevalence of COVID-19 was found to be comparable in HT patients and in the general population, suggesting comparable susceptibility (7). In heart transplant (HT) recipients, vaccination against COVID-19 is associated with a lower risk of infection [hazard ratio (RR), 0.41; 95% CI, 0.30–0.56], hospitalization (RR, 0.29; 95% CI, 0.14–0.61) and death (RR, 0.19; 95% CI, 0.05–0.82), with no transplantation-specific adverse events (8, 9). The development of a vaccine requires knowledge of what constitutes protective immune response. Circulating neutralizing SARS-CoV-2 antibodies have been shown to be a major contributor to protective immunity. They are providing attenuated disease severity (10) and are potentially even critical for survival (11). However, neutralizing antibody levels have been increased regarding new variants of SARS-CoV-2. Omicron sublineages, BA.4 and BA.5, are now dominant and these circulating variants of SARS-CoV-2 can substantially escape neutralizing antibodies induced by vaccination or previous infection (12–14).

CD4^+^ and CD8^+^ T-cell responses have also been shown to play important roles in the resolution of SARS-CoV-2 infection (15) and the modulation of COVID-19 severity (16). While coordinated CD4^+^ T cell, CD8^+^ T cell, and antibody responses are protective, uncoordinated responses frequently fail to control disease (16). SARS-CoV-2-specific CD8^+^ T-cell response is an important determinant of immune protection in mild SARS-CoV-2 infection (17). On the contrary, functional defects in CD4+ T cells and exhaustion of CD8+ T cells are associated with severe COVID-19 outcomes (18). It is recognized that immunosuppressed patients such as transplant recipients tend to have a blunted immune response to vaccines. In previous studies, T cell response was measured 2–3 weeks after vaccination in small groups of HT subjects, the proportion of positive T-cell responders seems to increase with the number of doses (19–21). A protective virus neutralization titer level is not yet established and the level of efficient T-cell response to induce protection after vaccination is unknown. Currently, data on the efficacy of SARS-CoV-2 vaccination among HT recipients are limited, in particular for T-cell response. We thus aimed to analyze the humoral and cellular response to vaccination after several doses of a COVID-19 mRNA vaccine on a longer period after vaccination in HT recipients taking immunosuppressive therapy, and to evaluate the factors associated with T-cell reactivity.

## Materials and methods

### Study participants

The study was conducted from July 27, 2021, to January 11, 2022. HT recipients who had been vaccinated against COVID-19 with mRNA-based vaccines (BNT162b2, Pfizer-BioNTech vaccine, for 90% of available data) were included. One patient refused any vaccination and was therefore excluded from the analysis. Three patients who refused the third dose were included in the two-dose group. A total of 96 patients underwent a single IgG and IFN-γ release assay testing during hospitalization or clinical follow-up.

Blood samples collected at varying times from 1 week to 7 months after vaccination were analyzed. Because patients were included throughout the study period, the number of doses were variable; the occurrence of a COVID-19 infection was pointed out. The institutional review board (Comité de Protection des Personnes Est I, Dijon) approved the protocol, and considered it to constitute routine clinical practice. The need for informed consent was waived, but all patients were given clear information about the study, and their non-opposition was obtained. Collection of nominative data was approved by the national authority for the protection of privacy and personal data.

### T-cell activity: IFN-γ release assay

The SARS-CoV-2-specific T-cell response was measured with a commercially available IFN-γ Release Assay (QuantiFERON starter CE-IVD, Qiagen) in heparinized whole blood following the manufacturer’s description.

Whole blood was stimulated with SARS-CoV-2 spike antigens during incubation for 16–24 h using two combination of peptides: Ag 1, covering the sub-unit S1 of the spike protein that stimulate CD4^+^ T cells and Ag 2, covering the S protein that stimulate both CD4^+^ and CD8^+^ T cells. Briefly, four blood collection tubes were drawn: Ag1, Ag2, and controls (nil and mitogen). One milliliter of whole blood was incubated for 16–24 h at 37°C, after which plasma was harvested and tested using an enzyme-linked immunosorbent assay (ELISA) for the presence of IFN-γ produced in response to the peptide antigens or mitogen. The nil result was adjusted for background, heterophile antibody effects or non-specific IFN-γ in blood samples. The results were expressed in IFN-γ IU/mL after subtraction of the negative control values as interpolated from a standard calibration curve. The cut-off was 0.15 IU/mL, as determined by the manufacturer.

### Humoral response: Anti-spike severe acute respiratory syndrome coronavirus 2 IgG antibodies

The SARS-CoV-2 IgG II Quant assay is a chemiluminescent microparticle immunoassay that is used for the quantitative determination of IgG antibodies, including neutralizing antibodies, to the receptor binding domain (RBD) of the subunit 1 of the S protein of SARS-CoV-2, using the Architect system (Abbott). Data were expressed in arbitrary units (AU)/mL. Binding antibody units (BAU/mL) were calculated after multiplication by 0.142 on the basis of the results of the World Health Organization International Standard study.^1^ The cut-off value was 50 AU/mL (equal to 7.1 BAU/mL), as determined by the manufacturer. A SARS-CoV-2 IgG concentration of 4,160 AU/mL (equal to 590 BAU/mL) considered to be neutralizing at 95% with the ancestral strain (SARS-CoV-2 IgG II Quant Assay User Manual, Abbott Laboratories, Diagnostics Division, 2020), was defined as high in this study.

### Statistical analysis

Continuous variables were expressed as means ± SEM, and categorical variables as frequencies and percentages. Univariate regression analysis was used to assess the relationship between having Ac > 590 BAU/mL and a positive T-cell response. To study variables associated to T-cell response, two major multivariate regression models were applied to ensure the reliability of the variables we finally selected.

The multivariable fractional polynomials (MFP) logistic regressions model was used to investigate non-linear associations between T-cell response and selected variables. Correlations between variables were checked before inclusion in the model in order to avoid collinearity. The MFP model is a method that allows software to determine whether an explanatory variable is important for the model (22–24). Because of the relatively small sample size of our study, we applied bootstrapping for internal validation (bootstrap resampling 200 times) to confirm the model.

The second multi-logistic regression model we used was the least absolute shrinkage and selection operator (LASSO). LASSO regression analysis with EBIC (extended Bayesian information criterion) was performed to achieve enhanced variable selection (25, 26). Compared with other linear regressions, LASSO is more applicable for the analysis if complex multicollinearity data because it minimizes insignificant coefficients to 0 (27). Statistical significance was set at *p* < 0.05. STATA v15 (StataCorp LLC, College Station, TX, USA) was used for the statistical analyses.

## Results

### Patients characteristics

Among the 96 recipients included in the study, 95 had a heart transplantation and one had a heart and kidney transplantation. The cohort included a high percentage of male patients (83.3%) with an average age of 58.7 ± 1.28. A vast majority of the patients were older than 40 (91%).

The time since transplant was variable, ranging between < 1 and 31 years before study inclusion (median 6.6 years). The major reasons for transplant were dilated cardiomyopathy (39.2%) and ischemic heart disease (33%). Half of the patients had a high BMI (>25), and half were active smokers. Almost all patients (97%) were on an immunosuppressive regimen with prednisolone, and about half (45%) were on mycophenolate mofetil (MMF) combined with an mTOR inhibitor (everolimus or sirolimus). Previous medical history included hypertension (*n* = 26) and diabetes (*n* = 15); 13 patients had a family history of coronary heart disease (Supplementary Table 1).

Patients were mostly vaccinated with mRNA vaccines, except for one patient who received only the chAdOx1-S recombinant vaccine (Astrazeneca), four patients received heterologous vaccination with mRNA and recombinant vaccine, one in the two doses group, four in the three doses group (Table 1). Data on the vaccine type were missing for five other patients. Seven of the transplant patients have had clinical SARS-CoV-2 infection prior to the first vaccination dose, two patients were infected between the second and the third dose, one each with the Alpha (B.1.1.7) and Beta (B.1.351) variants. All remained moderately clinically infected. COVIDs severity were classified according to the guidelines by medical physicians.^2^ Although about three quarters (74%) of patients had lymphopenia, 27 (28.1%) patients had a positive result for Ag1 and 39 (40.6%) had a positive Ag2 (IFN-γ ≥ 0.15 IU/mL) (Supplementary Table 2). A positive IgG response (IgG > 7.1 BAU/mL) was obtained for 73.4%, and 22 patients had antibodies > 590 BAU/mL (23.4%).

**TABLE 1:** Vaccination.

mRNA COVID-19 vaccine		
BNT162b2 (Pfizer/BioNtech)	76	79.2%
mRNA-1273 (Moderna)	9	9.4%
BNT162b2 + mRNA-1273	1	5.2%
BNT162b2 + chAdOx1-S	4	
chAdOx1-S recombinant (Astrazeneca)	1	1.0%
Non available data	5	5.2%
**Number of doses**		
1	2	2.1%
2	15	15.5%
3*	71	73.2%
4*	8	8.2%

3* For two patients: infection + 2 doses. 4* For seven patients: infection + 3 doses.

Patients had more neutrophils in the smoker group compared to the non-smoker group (smoker group: mean 4.92 ± 0.24; non-smoker group: mean 4.92 ± 0.24, *p* < 0.01 by Kruskal-Wallis test), however, analysis with logistic regression showed that neutrophils count was not a significant predictor of a positive T-cell response in univariate analysis.

Patients positive for Ag1 were all positive for IgG. Among the 96 enrolled patients, about one third were positive for Ag1, Ag2, and IgG, *n* = 32 (36.2%), 22 of them had IgG > 590 BAU/mL.

T-cell responses were detected in 20% (5/25) of seronegative subjects.

Seropositive responses were detected in 63.6% (35/55) of negative T cell subjects, but among these, 7.2% (4/55) had antibodies at a high level (>590 BAU/mL).

Most of the patients with IgG > 590 BAU/mL were T responders [81.8% (59.7–94.8%)]. Analysis with logistic regression showed that Ac > 590 BAU/mL was an significant predictor of a positive T-cell response in univariate analysis (*p* = 0.000).

### Factors independently associated with T-cell activity in heart transplant patients

We conducted a review of the literature to construct two predictive models and to ensure that crucial variables were not omitted from the prediction model. To avoid over-fitting of the model, we included 10 variables in the MFP analysis: age, sex, and variables reported as potentially related to immune response after vaccination (number of doses, occurrence of a SARS-CoV-2 infection, active smoking, MMF plus everolimus treatment, MMF plus cyclosporine, IgG response, the time lapse between last dose and dosage, and the lymphocyte count). Multivariable analysis using MFP revealed that each Ag1 and Ag2 response was independently associated with: age (Ag1 *p* = 0.011; Ag2 *p* = 0.034), the IgG response (Ag1 *p* = 0.000; Ag2 *p* = 0.000), the time elapsed since the last vaccine dose (Ag1 *p* = 0.048; Ag2 *p* = 0.013), the occurrence of a SARS-CoV2 infection (Ag1 *p* = 0.001; Ag2 *p* = 0.003), and the lymphocyte count (Ag1 *p* = 0.018; Ag2 *p* = 0.037). In addition, active smoking tended to be significant (Ag1 *p* = 0.060; Ag2 *p* = 0.063). We did a bootstrap for internal validation, the results of the MFP and the bootstrap were reliable, except for IgG response, which was no longer significantly associated. Active smoking was significantly associated with IFN-γ release after bootstrapping (Ag1 *p* = 0.039; Ag2 *p* = 0.047). The adjusted R-squared value of MFP model was 0.683, meaning that the significant variables explained 68.3% of the T-cell response (Table 2).

**TABLE 2:** Analysis of factors associated with T-cell response.

		MFP		MFP, bootstrap 200
**AG1 (CD4)**	0.26 ± 0.06	***R*^2^ = 0.683**		**adj *R*^2^ = 0.640**
Variables		*p*-value		*p*-value
Ig G anti-Spike	725 ± 198	**<0.0001**		ns
Number of doses		ns		ns
Sex (Male-%)	80 %	ns		ns
Age	58.7 ± 1.28	**0.011**		**0.024**
Time lapse vaccine-dosage (months)	3.8 ± 0.19	**0.048**		ns
**Occurrence of a SARS-CoV-2 infection**	*n* = 9	**0.001**		**0.005**
Lymphocyte count	1.2 ± 0.06	**0.018**		**0.046**
**Active smoking**	43.3%	0.060		**0.039**
MMF plus everolimus	33.3%	ns		ns
MMF plus cyclosporin	18.8%	ns		ns

		**MFP**	**MFP, bootstrap 200**	**Lasso2**

**AG2 (CD4+CD8)**	**0.39 ± 0.08**	***R*^2^ = 0.683**	**adj *R*^2^ = 0.640**	**adj *R*^2^ = 0.516**

Variables		*p*-value	*p*-value	*p*-value
Ig G anti-spike	725 ± 198	**0.0001**	ns	
Number of doses		ns	ns	**0.015**
Sex	80 %	ns	ns	ns
Age	58.7 ± 1.28	**0.034**	**0.037**	ns
Time lapse vaccine-dosage (months)	3.8 ± 0.19	**0.013**	ns	**0.023**
**Occurrence of a SARS-CoV-2 infection**	*n* = 9	**0.003**	**0.016**	**<0.0001**
Lymphocyte count	1.2 ± 0.06	**0.027**	**0.049**	
**Active smoking**	43.3%	0.063	**0.047**	**0.030**
MMF plus everolimus	33.3%	ns	ns	ns
MMF plus cyclosporin	18.8%	ns	ns	ns

Continuous variables were expressed as means ± SEM and categorical variables as percentages. *p*-values < 0.05 are indicated in bold, *ns* non-significant.

Regarding Ag2, we used a supplemental statistical model, the LASSO2, to obtain more accurate results. The LASSO2 method was used to screen preliminary variables. Among the 10 variables included in MFP model, IgG response, age, lymphocyte count, MMF plus everolimus treatment, and MMF plus cyclosporine treatment were not selected by LASSO. The five remaining selected variables were sex, the time lapse between last dose to QFT dosage, number of doses, active smoking and occurrence of a SARS-CoV-2 infection.

According to the results of the regression analysis based on LASSO2, the number of doses (*p* < 0.015) was associated with T-cell activity (Ag2) in vaccinated patients with HT as well as the time lapse between dosage and vaccination (*p* < 0.023), the occurrence of an infection (*p* < 0.000) and smoking (*p* < 0.03). However, sex was not independently associated with Ag2 (*p* = 0.07). The adjusted R-squared value of the regression based on LASSO2, was 0.52, which means that 52% is the fraction of the T-cell response that is predicted by these independent variables (Table 2).

To sum up, the variables consistently selected with MFP, MFP bootstrapping and LASSO2 statistical models indicate that the variables independently associated with T-cell activity (Ag2) in vaccinated HT patients are occurrence of an infection and active smoking.

### Specific IgG and T-cell response according to number of doses

**FIGURE 1:**
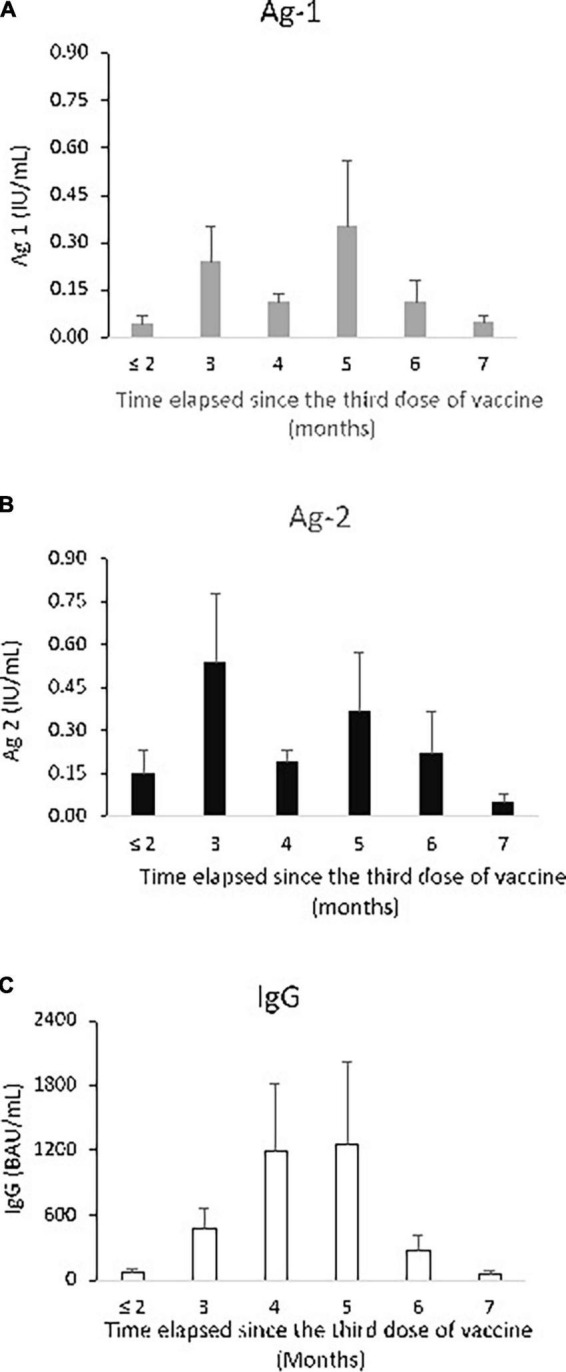
Specific T-cell and humoral responses after three doses, according to time elapsed since vaccination. Specific T-cell response as stimulated IFN-γ release: **(A)** QuantiFERON response to antigen 1, **(B)** QuantiFERON response to antigen 2, given in IU/mL. **(C)** Serological responses: Anti-SARS-CoV-2 IgG response, given in binding antibody units per mL (BAU/mL), at month ≤ 2 to month 7 after vaccination. The three dosages were done in the same Heart transplant patients [*n* = 9–20 per group, except for month 7 (*n* = 5)]. Mean ± SEM are indicated.

We first focused on patients that had received three doses of vaccine (*n* = 71). Mean values for T-cell response (Ag1 and Ag2) were above threshold from 3 to 5 months following the vaccine (mean of 0.20 0.06 for Ag1 and 0.31 ± 0.16 for Ag2) (Figure 1). Half of the patients had a positive T cell response (47.4%, 18/38), antibodies were detectable in a majority of patients (83.3%, 30/36). The humoral and cellular response to SARS-CoV-2 spike antigens peptides followed the same pattern, with immune response waning from 6 months post vaccination (Figure 1). In the two-dose group, mean T-cell response was lower than in the three-dose group and below the threshold (mean Ag1 = 0.03 ± 0.03; mean Ag2 = 0.09 ± 0.05). Some HT patients who had received a third dose of the vaccine had neither a humoral nor a cellular response at a time-lapse of 3–5 months after the last vaccination (11.1%, 4/36).

None of the patients from the two-dose group had high levels of antibodies (>590 BAU/mL). In the three-dose group, high antibodies levels were detected in 36.1% of the patients from 3 to 5 months after the last dose (13/36). In the small group 4including seven patients who had had three doses plus COVID-19 infection and one patient that received with four doses, all patients had high antibodies levels and positive T-cell response regardless of the time since the last vaccine dose (1–5 months).

Thus, a third vaccine dose generated a great anti-vaccine response in HT patients from 3 to 5 months post vaccination, and it also provided a proportion of nearly 50% of humoral and cellular responders.

**FIGURE 2:**
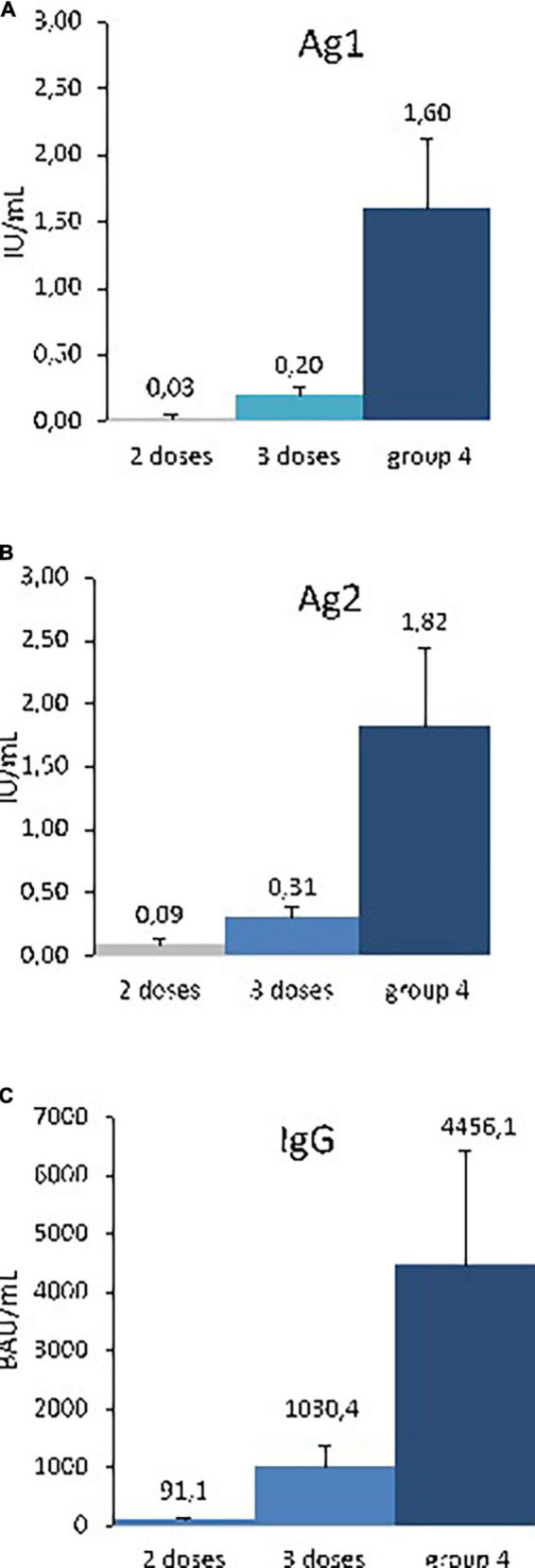
Specific T-cell and humoral responses after 2–4 doses, 3–5 months after vaccination. Specific T-cell response as stimulated IFN-γ release: **(A)** QuantiFERON response to antigen 1, **(B)** QuantiFERON response to antigen 2, given in IU/mL. Serological response **(C)** Anti-SARS-CoV-2 IgG response, given in binding antibody units per mL (BAU/mL), at months 3–5, after 2–4 doses of vaccination. The three dosages were done in the same Heart transplant patients (group 2 doses, *n* = 8; group 3 doses, *n* = 38, group 4, *n* = 5. Mean ± SEM are indicated. *p <* 0.01 for Ag1, Ag2, and IgG 2, comparaison between doses by Kruskal-Wallis test.

Immune response was far stronger in the group 4, probably because supplemental stimulation occurred via the natural infection for 7 out of 8 patients. From 3 to 5 months post vaccine, the mean IgG level reached 4456.1 BAU/mL, and mean Ag2 was 1.82 IU/mL (Figure 2).

## Discussion

Our investigation of immune response to mRNA vaccine in a large cohort of HT patients in the post vaccination period showed that humoral response was elicited more frequently than T-cell response.

COVID-19 severity varies widely, ranging from asymptomatic infection to severe pneumonia and multi-organ system dysfunction that can lead to the death. Overall, we reported a T cell response in 40.6% of HT patients, and nearly half of the HT patients who had received a third dose of the vaccine had a positive cellular response at a time-lapse of 3–5 months after the last vaccination. In previous studies, T cell response in HT subjects was measured only shortly after vaccination (2–3 weeks) (19–21). It was shown that most HT patients did not exhibit T-cell response, after the first and second doses (21) but the proportion of T-cell response was found to increase to 50–75% few weeks after the third dose and the fourth dose in a small group of patients (19, 20).

In previous studies, after two vaccine doses, the humoral immune response to the BNT162b2 vaccine was found to wane over a period of 6 months in HT patients (28, 29). We found a similar lower humoral response against SARS-CoV-2 after 5–6 months, even after three doses of BNT162b2. Most (90%) of the HT patients did not show a detectable humoral response after two doses of BNT162b2 (Pfizer) vaccine (25), 64% after mRNA-1273 (Moderna) vaccination (30) and 36% after ChAdOx1 nCoV-19 (AZD1222) (AstraZeneca) vaccination (31). We detected a seropositive response after two doses in 50% of subjects, this proportion increased to 77% after three doses and from 3 to 5 months, a high level of antibodies was seen. Our findings are in line with two recent studies. They reported, 3 weeks after vaccination, that additional third and fourth boosters of BNT162b2 vaccine improved the magnitude of IgG anti-RBD SARS-CoV-2 antibody response in seropositive HT recipients and the proportion of IgG responsive patients increase to 57 and 80%, respectively, compared to the range achieved after the two primary doses (19, 20, 32). Moreover, in our study, a large proportion of patients, more than eighty percent, with a high antibody response had a positive T-cell response. Coordinated T and B cell and antibody responses have been shown to be protective (16), and we showed here that a strong serological response (>590 BAU/mL) may be predictive of a good T-cell response in HT patients. Considering our observation that IgG and T-cell activity and IgG decreased sharply at 6 months, a booster dose of mRNA vaccine following the third immunization should be considered from 5 to 6 months, at least in immunocompromised populations. Duration of immune responses after heterologous vaccine regimens in HT recipients is unknown, however, heterologous primary SARS-CoV-2 immunization with ChAdOx1 and BNT162b2 has been shown to elicit a stronger initial humoral and T cell immune response compared to homologous vaccination with ChAdOx1 or BNT162b2 among health care workers (HCWs). Differences in humoral responses remain over 6 months, however, the SARS-CoV-2 specific T-cell responses were no longer significant 3 months after vaccination (33, 34).

A surprising finding of our study was that active smoking was statistically associated with quantitative T-cell response, which has not been previously reported. Many studies reported that smokers appeared to be protected against SARS-CoV-2 infections, while other studies underlined an increased risk for severe COVID-19 in heavy smokers. The effect of cigarette smoking on COVID-19 is still controversial (35) and the level of smoking may influence the conclusion (36). Unfortunately, we did not quantify the levels of smoking. Active smoking has been shown to negatively impact humoral response to COVID-19 vaccines in HCWs (37–41) although other studies found no significant association between smoking and anti-spike IgG levels (42, 43). In future, external validation studies are required to confirm the positive impact of smoking on T-cell response.

Not as reported for the humoral response in HT recipients (20), and more specifically to the dose of mycophenolate in lung transplants and HTs (44, 45), we did not find any statistical association between immunosuppressive treatment with MMF and T-cell response.

Our study also underlines the good immune response induced by infection in the small group of patients with a COVID-19 infection plus three doses, since all individuals had high IgG and T-cell response. Previous COVID-19 infection enhanced also magnitude and longevity of the humoral response following vaccination with mRNA vaccines of adult patients on hemodialysis or SOTs (46, 47). Similarly, SARS-CoV-2 infection prior to vaccination of HCWs resulted in substantially higher peak geometric mean titer and IFN-γ levels, and enhanced SARS-CoV-2 specific antibody and T cell responses over time (33, 48). The infection preferentially induces T cells that cross-recognize SARS-CoV-2/common cold coronaviruses rather than T cells induced by vaccine that are only specific for SARS-CoV-2 antigens. However, vaccination maintain the overall number of clones induced by prior infection and through induction of new clones, diversifies the repertoire (49–52). All the current mRNA vaccines were designed on the base of the ancestral strain of SARS-CoV-2 spike protein. Newer SARS-CoV-2 variants have numerous mutations on the S protein leading to humoral escape, but T-cell response has been shown to be preserved in convalescent donors and vaccines (53–55). This highlights the importance of actively monitoring T-cell reactivity, in particular in immunosuppressed patients.

Having 3 vs. 2 or zero doses of mRNA vaccine was associated with increased protection of the general population against both the Omicron and Delta variants (56). Booster vaccination with mRNA vaccines maintains over 70% protection against hospitalization and death in breakthrough confirmed omicron infections (57). Vaccination, primary infection and also breakthrough infection (BTIs) constitutes repeated antigen exposures and all elicit diverse repertoires and diversify the T cell memory (58). In a recent review, BTIs due to the variant delta were shown to be more common in immunodeficient individuals and HCWs than in healthy individuals. Immunosuppressed individuals were more likely to be hospitalized after infection, however, no specific variant was associated with severe disease and the majority of patients recovered (59, 60). A better knowledge of immunity after vaccination and BTI in fragile populations is essential to face emerging variants of SARS-CoV-2. Moreover, a third dose of BNT162b2 vaccine has been recommended for solid organ transplant recipients by the French Administration since August 6, 2021 and new vaccines adapted to BA4 and BA5 variants are coming.

Some side effects of SARS-CoV-2 vaccines, such as fever, headache, fatigue and pain at the injection site have been described. Organ rejection post-COVID-19 vaccination is rare but can occur with all SARS-CoV-2 vaccines or following COVID-19 infection. However, only one case of heart rejection among 136 cases was described by Alhumaid et al. (61). The small number of reported case and the protective benefits of vaccination against SARS-CoV-2 especially for transplant patients should not discourage vaccination (9).

We performed T cell analysis using QuantiFERON assay, that does not need to isolate PBMC. The same volume of blood is drawn, the T cell response is evaluated by mL of blood and can be compared between patients. Alternatively, cytokine-producing cells may be enumerated using an ELISPOT assay or flow cytometry-based intracellular cytokine staining. ELISPOT gives the IFN-γ cell concentration per T cell. In the context of tuberculosis disease (TB), the QFT-TB assay lacks of sensitivity with many indederminate results. These are related to an alteration of the mitogen response (62) and are encountered in patients where T-cells exhaustion is frequent. Comparative sensitivity and specificity analysis of T-cell assays to SARS-CoV-2 is not available, however, we did not find any such indeterminate result for QFT SARS-CoV-2.

One limitation in our study is the heterogeneity of the group. Assays were done at the time of clinical follow up, and IgG and T-cell activity was assessed once rather than repeatedly, so we were not able to perform a longitudinal analysis. On the other hand, the post-vaccination study period was long enough to observe the waning of humoral and cellular immunity.

Overall, studies on T-cell response in HT are currently sparse. Our study provides novel data on the immunogenicity of COVID-19 mRNA vaccines on a large cohort of HT patients, in particular on T cell response. Our findings underline the interest of booster vaccinations among HT recipients, who are a population at risk of severe disease, and we suggest the importance of follow-up for antibody and T-cell response following vaccination in order to ensure sufficient immune protection.

## Data availability statement

The raw data supporting the conclusions of this article will be made available by the authors, without undue reservation.

## Ethics statement

Ethical review and approval was not required for the study on human participants in accordance with the local legislation and institutional requirements. The need for informed consent was waived, but all patients were given clear information about the study, and their non-opposition was obtained.

## Author contributions

J-BB, CA, and SG involved with study design. AC, SG, and CM contributed to patient data acquisition. L-SA-G and CM made the statistical analysis. CM wrote the manuscript. AC, J-BB, CA, L-SA-G, and SG revised the manuscript. All authors contributed to the article and approved the submitted version.

## Footnotes

1
https://www.who.int/publications/m/item/WHO-BS-2020.2403

2
https://www.covid19treatmentguidelines.nih.gov/overview/clinical-spectrum/

## Abbreviations

HT: Heart transplant
HCW: Health care workers
SARS-CoV-2: Severe Acute Respiratory Syndrome Coronavirus 2
RBD: receptor binding domain
COVID: coronavirus disease
BTI: breakthrough infection
BMI: body mass index
MMF: mycophenolate mofetil
mRNA: messenger ribonucleic acid
IFN-γ: Interferon-γ
Ag: Antigen
ELISA: Enzyme Linked ImmunoSorbent Assay
BAU: binding antibody units
AU: arbitrary units
QFT: QuantiFERON
MFP: multivariable fractional polynomials
LASSO: least absolute shrinkage and selection operator
EBIC: extended Bayesian information criterion
SEM: standard error of the mean.
