# Supplementary material for: Humoral and cellular immune response after severe acute respiratory syndrome coronavirus 2 messenger ribonucleic acid vaccination in heart transplant recipients: An observational study in France
Source: Front Med (Lausanne). 2022 Oct 26;9:1027708. doi: 10.3389/fmed.2022.1027708 (PMC9643719; doi:10.3389/fmed.2022.1027708)
Supplement: Supplementary file 1 [file Table_1.pdf]

Table S1: Characteristics of heart transplant patients (n=96)

Age – years	mean 58.7 ±1.28 (20-86)<br>median 61.7	
20-40	9	9.4%
41-60	36	37.5%
60+	51	53.1%
Sex: Male – n (%)	80	83.3%
Family history of coronary disease	13	13.5%
Arterial hypertension	26	27.1%
Diabetes – type 1 or type 2	15	15.6%
BMI – kg/m^2	mean 26 ± 0.48 (18-44.)<br>median 24.6	
<18.5	1	1.0%
18.5-24.9	48	50.0%
25-29.9	32	33.3%
30-39.9	13	13.5%
≥40	2	2.1%
History of smoking	11	11.3%
Active smoking	42	43.3%
**Type of organ transplantation**		
Heart	95	99.0%
Heart–kidney	1	1.0%
**Time since transplantation (years)**	mean 9.2 ± 0.77 (0.2-31.7)<br>median 6.6	
**Medical reason for transplant**		
Dilated cardiomyopathy	38	39.6%
Ischemic heart disease	32	33.3%
Hypertrophic cardiomyopathy	9	9.4%
Valvular heart disease	4	4.2%
Transplantation on rejection	3	3.1%
Anthracycline-related myocarditis	1	1.0%
Congenital heart disease	2	2.1%
Healthy coronary cardiomyopathy	1	1.0%
Arrhythmogenic dysplasia of the right ventricle	1	1.0%
Restrictive heart disease	1	1.0%
Dilated ischemic heart disease	3	3.1%
Valvular and ischemic heart disease	1	1.0%
**Type of maintenance immunosuppression**		
Prednisolone	94	97.9%
Cyclosporin/everolimus	27	28.1%
Cyclosporin/MMF (mycophenolate mofetil)	18	18.8%
MMF/ mTOR inhibitor everolimus	32	33.3%
MMF/ mTOR inhibitor sirolimus	11	11.5%
MMF /CNI tacrolimus	2	2.1%
Azathioprine/CNI	3	3.1%
Azathioprine/ everolimus	4	4.2%
