# Supplementary material for: Humoral and cellular immune response after severe acute respiratory syndrome coronavirus 2 messenger ribonucleic acid vaccination in heart transplant recipients: An observational study in France
Source: Front Med (Lausanne). 2022 Oct 26;9:1027708. doi: 10.3389/fmed.2022.1027708 (PMC9643719; doi:10.3389/fmed.2022.1027708)
Supplement: Supplementary file 2 [file Table_2.docx]

**Table S2 Biological results**

|  | Time between vaccination and QFT + serology (months) | QFT: **Ag1**  (UA/mL) | QFT: **Ag2**  (UA/mL) | **IgG**  (BAU/mL) | **Lymphocyte**  **count** (x10^3^/µL) | **Neutrophil**  **Count**  (x10^3^/µL) | **Eosinophil count** (x10^3^/µL) | **Basophil count** (x10^3^/µL) | **Monocyte**  **count** (x10^3^/µL) |
| --- | --- | --- | --- | --- | --- | --- | --- | --- | --- |
| **Patients**  (n=96) | mean 3.8 ± 0.19  (0.2-7.5)  median 3.9 | mean 0.26 ± 0.06 (0.06-2.96) median 0.05 | mean 0.39 ± 0.08 (0.01-3.13) median 0.08 | mean 725 ± 196.6 (0-11 360) median 61 | mean 1.2 ± 0.06 (0.25-3.02) median 1.1 | mean 4.46 ± 0.14 (2.82-8.81) median 4.12 | mean 0.1 ± 0.01 (0.02-0.45) median 0.09 | mean 0.03 ± 0.002 (0.02-0.15)  median 0.02 | mean 0.68 ± 0.02 (0.29-1.24)  median 0.66 |
| Age group |  |  |  |  |  |  |  |  |  |
| **20-40**  (n=9) | mean 5.11 ± 0.76 | mean 0.16 ± 0.12 | mean 0.3 ± 0.25 | mean 539 ± 256 | mean 1.09 ± 0.08 | mean 4.81 ± 0.51 | mean 0.09 ± 0.02 | mean 0.02 ± 0.004 | mean 0.63 ± 0.09 |
| **41-60**  (n=36) | mean 3.89 ± 0.27 | mean 0.44 ± 0.13 | mean 0.61 ± 0.16 | mean 891 ± 289 | mean 1.3 ± 0.11 | mean 4.46 ± 0.19 | mean 0.09 ± 0.01 | mean 0.02 ± 0.002 | mean 0.68 ± 0.03 |
| **60+**  (n=51) | mean 3.58 ± 0.27 | mean 0.14± 0.05 | mean 0.24 ± 0.0 | mean 641 ± 307 | mean 1.15 ± 0.07 | mean 4.39 ± 0.22 | mean 0.11 ± 0.01 | mean 0.03 ± 0.003 | mean 0.68 ± 0.03 |
